# p52 signaling promotes cellular senescence

**DOI:** 10.1186/s13578-022-00779-6

**Published:** 2022-04-04

**Authors:** Giovanna M. Bernal, Longtao Wu, David J. Voce, Ralph R. Weichselbaum, Bakhtiar Yamini

**Affiliations:** 1 Department of Neurological Surgery, The University of Chicago, Chicago, IL 60637 USA; 2 Department of Radiation and Cellular Oncology, The Ludwig Center for Metastasis Research, The University of Chicago, Chicago, IL 60637 USA

**Keywords:** p52, Senescence, STAT3, S100A4, CYPA, NF-κB

## Abstract

**Background:**

Nuclear factor-κB is a multi-subunit transcription factor that plays a central role in cellular senescence. We previously reported that an increase in the p52 subunit is seen in senescent cells and aged tissue. In the current work, we examined the mechanism by which p52 is activated and whether the increase in p52 promotes senescence.

**Results:**

Using both primary mouse embryonic fibroblasts (MEFs) and WI-38 human lung fibroblasts, we examined cells after serial passage and following prolonged culture. An increase in p52 was found in the nucleus relative to pre-senescent cells. The increase in p52 protein was not reflected by an increase in *NFKB2* mRNA or by an increase in the abundance of upstream activating kinases, IKKα and NIK. To examine whether p52 promotes senescence, we over-expressed mature p52 in primary MEFs. Significantly more senescence was seen compared to control, a finding not seen with p52 mutated at critical DNA binding residues. In addition, blocking p52 nuclear translocation with the peptide inhibitor, SN52, decreased β-galactosidase (β-gal) formation. Subsequent filtration studies demonstrated that proteins in conditioned media (CM) were necessary for the increase in p52 and mass spectrometry identified S100A4 and cyclophilin A (CYPA) as potential factors in CM necessary for induction of p52. The requirement of these proteins in CM for induction of p52 was confirmed using depletion and supplementation studies. In addition, we found that activation of STAT3 signaling was required for the increase in p52. Finally, genome wide ChIP-sequencing analysis confirmed that there is an increase in p52 chromatin enrichment with senescence and identified several downstream factors whose expression is regulated by increased p52 binding.

**Conclusions:**

These results demonstrate that p52 nuclear translocation is increased in senescent cells by factors in conditioned media and that mature p52 induces cellular senescence. The data are consistent with the prior observation that p52 is elevated in aged tissue and support the hypothesis that p52 contributes to organismal aging.

**Supplementary information:**

The online version contains supplementary material available at 10.1186/s13578-022-00779-6.

## Background

Initially described as a response to repeated passage, cellular senescence is now recognized as a process induced by a wide variety of cell intrinsic and extrinsic factors [1]. While early work focused on senescence as an *in vitro* phenomenon, it is clear that cellular senescence also occurs in living animal tissue. Seminal studies over the past decade have identified the critical role of cellular senescence in promoting organismal aging [2]. In addition, senescence has been shown to play a multi-faceted role in carcinogenesis. In this regard, whereas senescent removal of transformed and potentially malignant cells acts as a tumor suppressive process [3], release of a senescence associated secretory phenotype (SASP) induces inflammation that promotes tumor formation [4].

Despite the growing understanding of the mechanism by which senescent cells promote their paracrine effects, there remains a lack of knowledge regarding the molecular mechanisms by which senescence is induced. Nuclear factor-κB (NF-κB) is recognized as a master regulator of processes that mediate senescence [5]. Moreover, this transcription factor has been identified as a critical node in mammalian aging [6, 7]. While the majority of studies examining NF-κB and senescence/aging have focused on the p65 (RELA) subunit and canonical signaling, it is likely that other NF-κB subunits also contribute to this process. NF-κB is a ubiquitous transcription factor comprised of five primary subunits, p50 (NF-κB1, p105), p52 (NF-κB2, p100), p65, RELB and C-REL that dimerize in a variety of combinations to mediate their effects [8]. This diversity contributes to the complex nature of the NF-κB response and underlines the observation that findings with one subunit may not be relatable to others.

While the Rel subunits are produced in their mature form, the two non-Rel subunits, p52 and p50, are synthesized as precursor proteins. p52 is generated from its parental protein p100 in a tightly regulated manner [9, 10] that is controlled primarily by NF-κB-inducing kinase (NIK), a constitutively degraded protein [11]. A variety of stimuli, including signals emanating from surface receptor/ligand binding [11] or pathways induced by the innate immune response [12, 13], converge on NIK or its upstream regulators to induce p52 nuclear translocation [14, 15]. Although p52 has traditionally been linked to RELB and non-canonical NF-κB [16], this subunit can also dimerize with itself and other subunits [8]. It is important to appreciate that while p52 generally acts in a pro-inflammatory and carcinogenic manner, p100, its parental protein, is primarily cytoplasmic and has the opposite effect, acting as an apoptotic and tumor suppressive factor [15, 17, 18].

Although a general increase in NF-κB activity is well recognized as a prominent feature of aging [7, 19], subunit specific changes in nuclear NF-κB have not been extensively studied. In examining the NF-κB subunit profile of aged animals compared to young, we previously reported that one of the most profound changes in NF-κB with age was a substantial increase in the amount of mature p52 protein in aged tissue [20]. In the current work, we demonstrate that p52 is increased in the nucleus via proteins present in conditioned media and, using overexpression and depletion studies, demonstrate that p52 induces cellular senescence. The findings presented indicate that elevated mature p52 acts to promote cellular senescence and support the hypothesis that the elevated p52 found in aged tissue contributes to aging.

## Results

### Cellular senescence is associated with increased nuclear p52

**Fig. 1:**
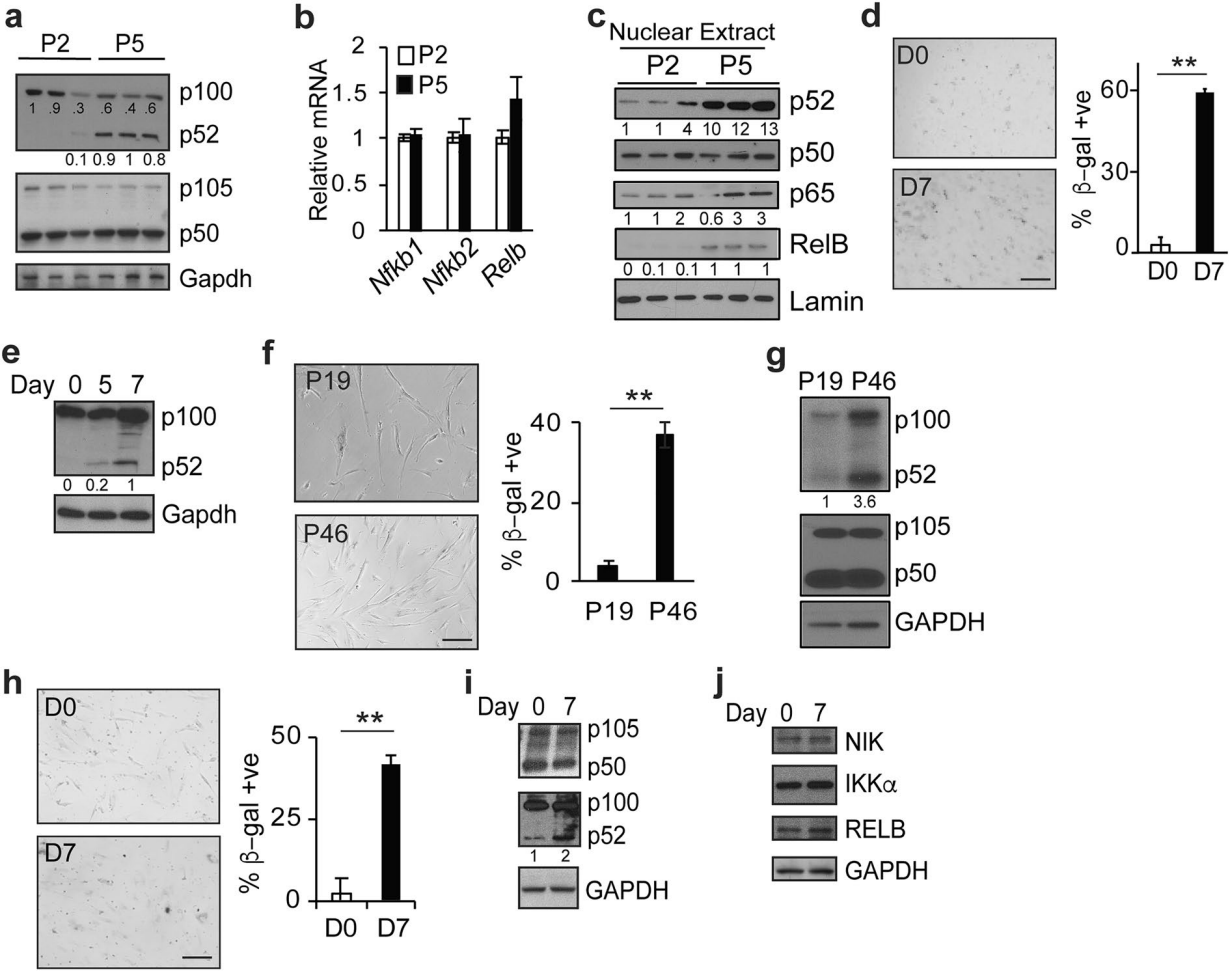
Cellular senescence is associated with increased nuclear p52. **a** Immunoblot (IB) using lysate from mouse embryonic fibroblasts (MEFs) at passage 2 (P2) and 5 (P5). IB performed with anti-p52, anti-p50, and anti-Gapdh. **b** qPCR analysis of indicated mRNA in MEFs at P2 and P5. Data show mean value of triplicate biological samples relative to *Gapdh*, ± SEM normalized to P2. **c** IB using nuclear extract from MEFs probed with indicated antibodies. **d** Quantification of β-gal staining of MEFs on Day 0 (D0) and 7 (D7). Representative images (left). Data show mean value of at least 200 cells per sample of three biological repeats, ± SEM. **e** IB probed with anti-p52 using lysate from MEFs at D0, D5, and D7. **f** Quantification of β-gal staining of WI-38 cells at P19 and P46. Representative images (left). **g** IB using lysate from WI-38 cells probed with the indicated antibodies. **h** Quantification of β-gal staining in WI-38 cells at D0 and D7. **i** and **j** IB using lysate from WI-38 cells at Day 0 and D7 probed with the indicated antibodies. Scale bar, 100 μm. ** *P* < 0.01 (two-tailed *t* test). Blots are representative of at least two biologically independent experiments. Analysis of fold-change normalized to control lane shown below IB where indicated

In our hands, serial passage of primary mouse embryonic fibroblast (MEFs) results in a significant increase in β-galactosidase (β-gal) staining by passage 5 (P5) that is accompanied by a change in morphology to an enlarged and flattened shape (Additional file 1: Fig. S1a). In conjunction with this, p52 abundance is substantially elevated at P5 compared to P2, a finding not seen with the p50 subunit (Fig. 1a). Notably, the increase in p52 is not associated with a change in *Nfkb2* mRNA expression in late passage MEFs (Fig. 1b). As NF-κB subunits mediate their effects in the nucleus, we examined nuclear fractions. Again, a substantial increase in p52, and Relb, protein is seen in the nucleus of late passage MEFs compared to early passage (Fig. 1c). We also examined the primary upstream regulators of p52 nuclear translocation, NIK and IKKα, and found a lack of consistent or substantial change in the abundance of either protein following serial passage (Additional file 1: Fig. S1b).

To further examine the link between p52 and senescence *in vitro*, we used a different model involving long-term cell culture. MEFs were continuously cultured for 7 days. By day 7, a significant increase in β-gal positive cells was seen (Fig. 1d). Moreover, examination of p52 demonstrates that its abundance also increased after several days in continuous culture (Fig. 1e). We next examined these changes in human cells. WI-38 human lung fibroblasts were serially passaged and a significant increase in β-gal staining was seen at passage 46 compared to passage 19 (Fig. 1f). As in MEFs, there was a concomitant increase in p52, but not p50, protein in late passage cells (Fig. 1g). In addition, similar to extensive passage, after 7 days in continuous culture WI-38 cells also have an increase in β-gal staining and p52 abundance (Fig. 1h and i) with no change in either NIK or IKKα protein (Fig. 1j). Together, these results indicate that replication-associated cellular senescence is associated with increased nuclear p52.

### p52 promotes cellular senescence

**Fig. 2:**
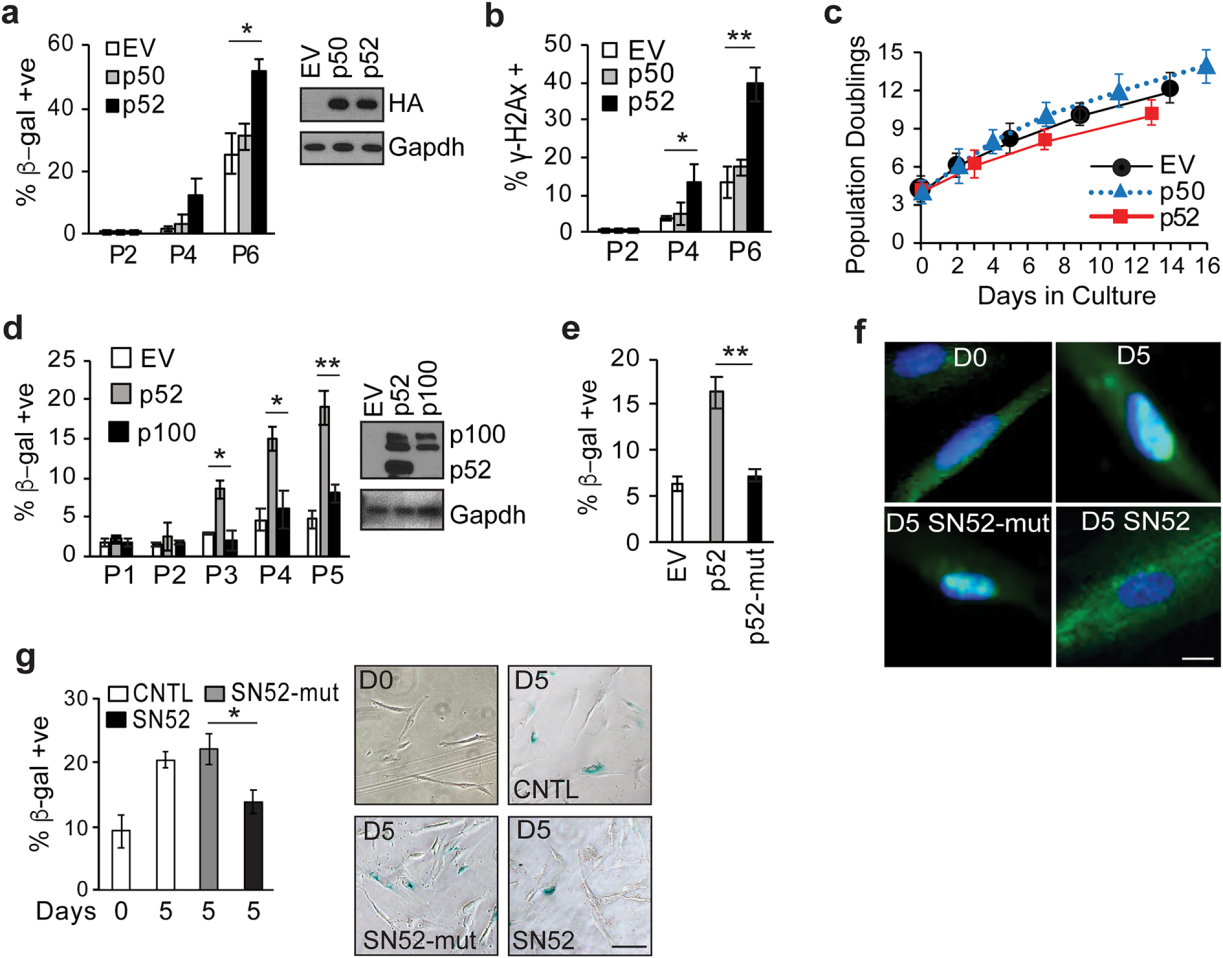
p52 promotes cellular senescence. **a** Quantification of β-gal staining of MEFs expressing empty vector (EV), HA-tagged p50 or p52 at indicated passage. Inset: Immunoblot (IB) with anti-HA. **b** Quantification of γ-H2AX foci staining in MEFs expressing indicated construct. **c** Growth curves of MEFs from experiment **a** at indicated passage following derivation (set as time 0). **d** Quantification of β-gal staining of MEFs expressing p52, p100 or EV. Inset: IB using lysate from cells expressing indicated construct at time of initial infection, probed with anti-p52. **e** Quantification of β-gal positive WI-38 cells expressing EV, wildtype p52 or p52-R54A/Y55A (p52-mut). **f** Representative immunofluorescence (IF) of endogenous p52/100 (green) in WI-38 cells on Day 0 (D0) and day 5 (D5) following treatment with vehicle, SN52 or SN52-mut. Counterstain with DAPI (blue). Scale bar, 10 μm. **g** Quantification of β-gal positive WI-38 cells on Day 0 and 5 following prolonged culture treated with vehicle (CNTL), SN52 or SN52-mut. Representative image shown (right); Scale bar, 100 μm. β-gal staining data represent mean value from at least 200 cells per sample from three biological repeats, ± SEM. * *P* < 0.05, ** *P* < 0.01 (two-tailed *t* test)

To examine whether p52 actually promotes senescence, we infected low passage primary MEFs with constructs containing mature p52 and then serially passaged these cells. While MEFs expressing p52 had an increase in β-gal positive cells following repeated passage compared to control, over-expression of p50 to a similar level as p52 did not alter the β-gal positivity (Fig. 2a). Notably, compared to empty vector (EV), over-expression of p52 also led to significantly greater γ-H2AX positive staining following serial passage (Fig. 2b). Also, examination of population doubling demonstrates that exogenous p52 significantly increased the time required for MEFs to double their number compared to p50 or EV (Fig. 2c). These results suggest that p52 promotes senescence and a decrease in cellular proliferation.

It was previously reported that knockdown of *NFKB2* promotes senescence [21]. This finding suggests that, contrary to our findings, p52 and or p100 inhibits senescence. We therefore wanted to examine p100 independently of mature p52 and therefore overexpressed both individually. To this end, MEFs were infected with constructs expressing either p100 or p52 and cells serially passaged. While p52 significantly increased the percentage of β-gal positive cells by passage 3, p100 did not (Fig. 2d) suggesting that these two proteins act in distinct ways. We next mutated p52 at two residues previously demonstrated to be required for DNA binding (R54 and Y55) [22] and examined β-gal in human WI-38 cells expressing this construct. While wildtype p52 significantly increased β-gal positive cell percentage, p52-R54A/Y55A (p52-mut) did not (Fig. 2e). These results indicate that mature p52, not its parental protein p100, induces cellular senescence and that DNA binding is required for p52 to promote senescence.

To further examine the role of p52, we wanted to study a loss-of-function model that inhibits p52 without reducing p100. To this end, we employed the specific peptide inhibitor, SN52, that blocks p52 nuclear translocation without altering p100 expression [23, 24]. Importantly, results with SN52 can be directly compared to its peptide control, SN52-mut. In our system, SN52, but not SN52-mut, significantly blocks p52 nuclear translocation as demonstrated by immunofluorescence (IF) imaging (Fig. 2f). Treatment of WI-38 cells with SN52, but not SN52-mut, significantly decreased the percentage of β-gal positive cells following continuous culture for 5 days (Fig. 2 g). Taken together, these results indicate that nuclear p52 promotes cellular senescence.

### Secreted factors promote p52 nuclear translocation

**Fig. 3:**
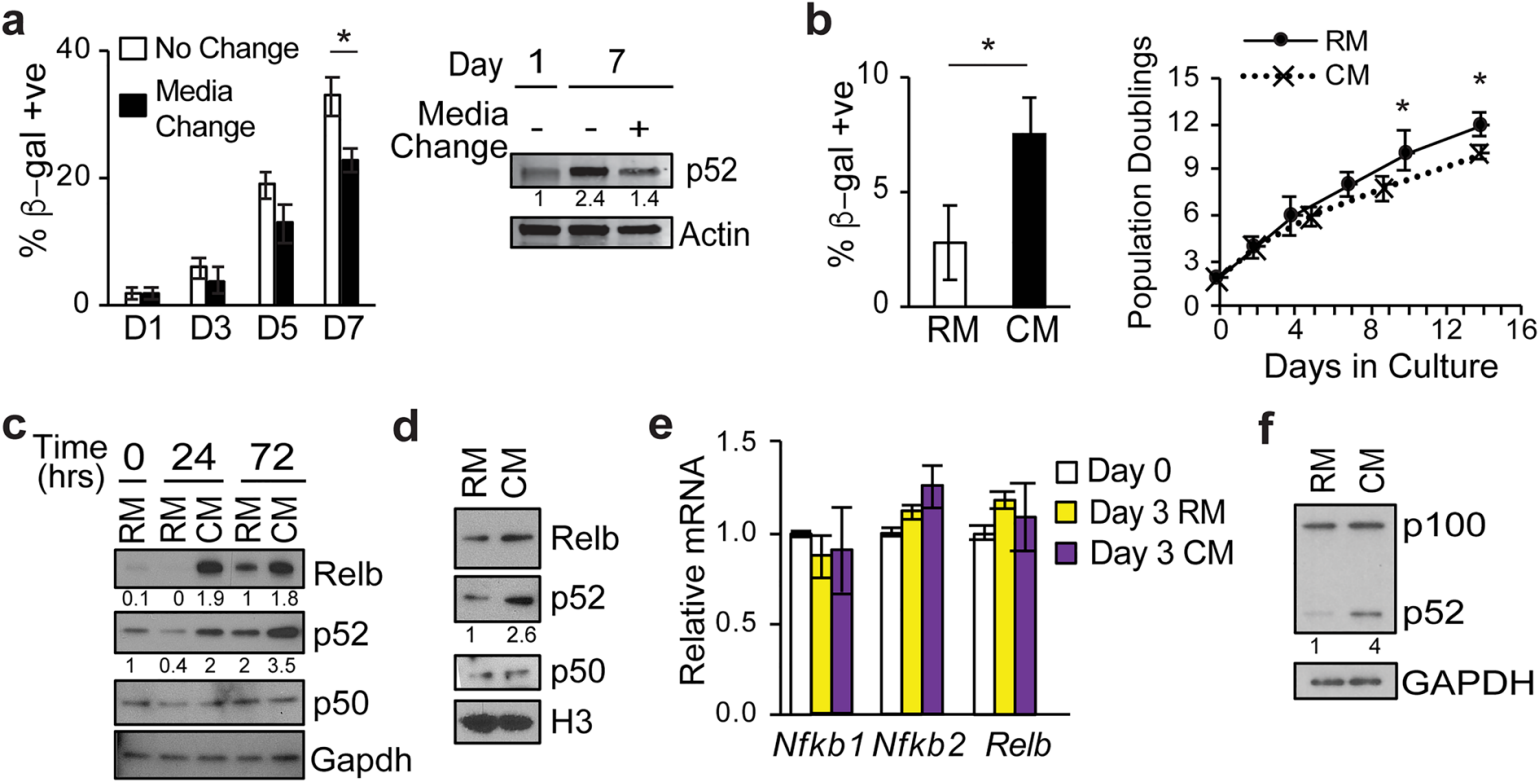
Conditioned media promotes p52 nuclear translocation. **a** Quantitative analysis (left) of β-gal-positive MEFs at indicated day following culturing with or without media change. Immunoblot (IB) (right) of same MEFs on Day 1 and 7 probed with anti-p52 and anti-Actin. **b** Quantification of β-gal-positive MEFs (left) on Day 3 following incubation with regular media (RM) or conditioned media (CM). Growth curves (right) of MEFs incubated in RM or CM passage 3 set as time 0. **c** IB using lysate from MEFs incubated in RM or CM for the specified time (hrs), probed with indicated antibodies. **d** IB using nuclear extract from MEFs incubated in RM or CM for 24 h. **e** qPCR analysis of mRNA from MEFs on Day 0 and 3 following incubation in RM or CM. Data show mean value of biological triplicate samples relative to *Gapdh*, ± SD normalized to Day 0. **f** IB with anti-p52 using lysate from WI-38 cells exposed to either RM or CM for 24 h. All data except qPCR represent mean value of three biological samples, ± SEM. *P < 0.05, **P < 0.01 (two-tailed *t* test). Blots are representative of at least two biologically independent experiments. Analysis of fold-change normalized to control lane shown below IB where indicated

The increase in nuclear p52 following prolonged culture suggested that factors present in the media contribute to the effect. To examine this hypothesis, we replaced the conditioned media (CM) with fresh media and examined p52 and β-gal staining. Replacement of CM with fresh media significantly decreased the percentage of cells that were β-gal positive and also reduced the increase in p52 protein seen with prolonged culturing (Fig. 3a), suggesting that factors in the CM induce p52 accumulation. We next harvested CM from MEFs and incubated early passage cells with this media. In the presence of CM there was significantly increased β-gal staining and increased population doubling time compared to MEFs incubated in regular media (RM) (Fig. 3b). Moreover, incubation in CM resulted in substantially increased p52 and Relb protein compared to MEFs incubated in RM (Fig. 3c), a finding not seen with p50. Of note, while p52 and Relb increase at 72 h in continuous culture even in RM, this increase is substantially augmented in the presence of CM. In addition, cell fractionation demonstrates that incubation in CM for even 24 h increased nuclear p52 and Relb (Fig. 3d). Given the increase in p52 protein, we examined whether this was associated with a concomitant change in *Nfkb2* mRNA. Total mRNA was isolated from MEFs in the presence of RM or CM and the expression of various NF-κB factors examined. No significant change in *Nfkb2* or *Relb* mRNA was seen in the presence of CM compared to RM (Fig. 3e). Similarly, no significant change in *NIK* or *IKKα* mRNA was seen (data not shown). We also examined WI-38 cells and found that, as with MEFs, CM also induced a robust increase in p52 protein in the human cells (Fig. 3f). These results indicate that secreted factors induce an increase in nuclear p52 and that the increase in p52 is not due to changes in mRNA expression.

### Extracellular CYPA and S100A4 induce p52

**Fig. 4:**
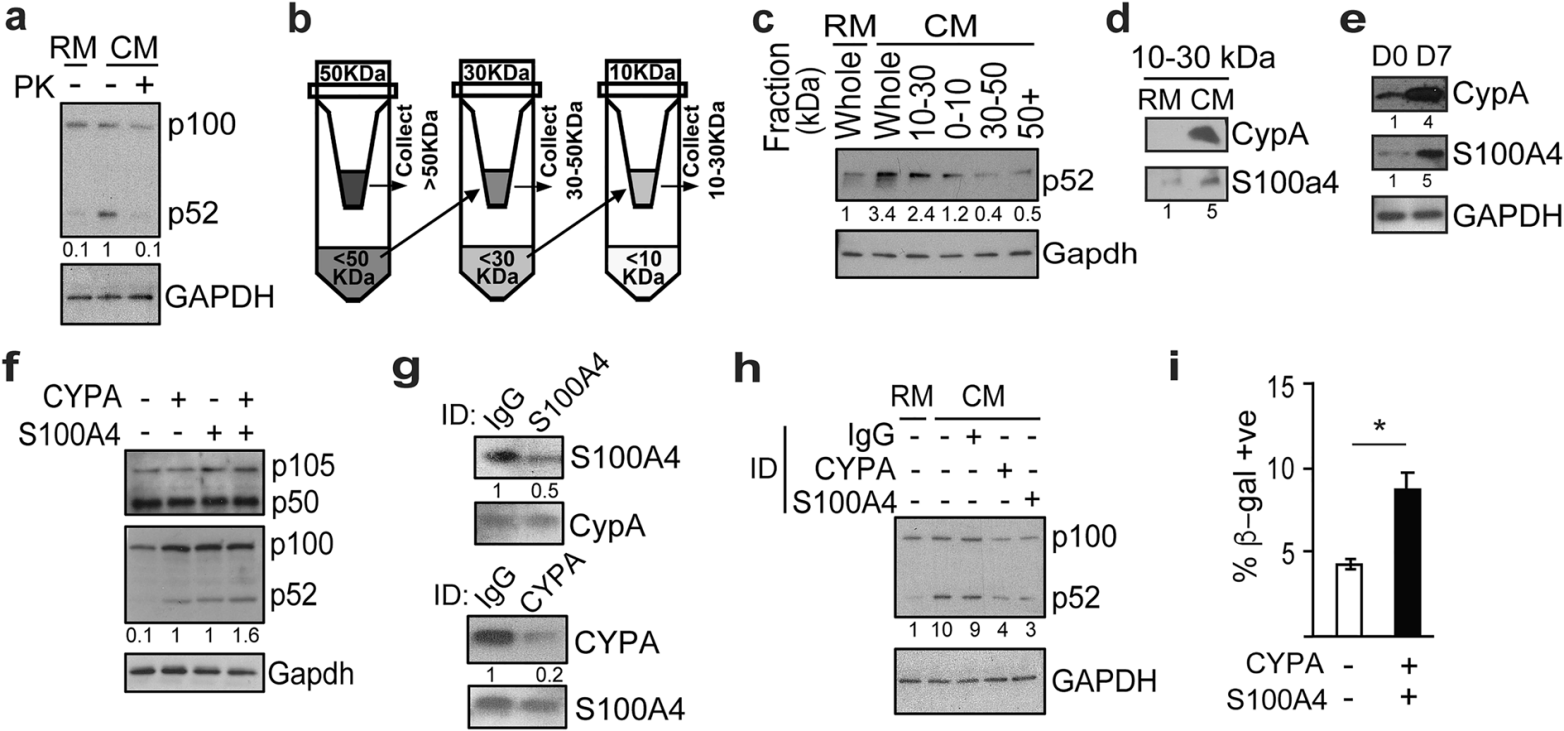
Extracellular CYPA and S100A4 induce p52. **a** Immunoblot (IB) with anti-p52 and anti-GAPDH using lysate from WI-38 cells incubated in regular media (RM) or conditioned media (CM) pre-treated with proteinase K (PK). **b** Schematic of CM fractions partitioned based on molecular weight. **c** IB with anti-p52 using lysate from MEFs incubated for 24 h in either whole RM (Whole) or the specific CM fraction indicated. **d** IB using the 10–30 kDa fraction isolated from RM or CM probed with the indicated antibody. **e** IB using lysate from WI-38 cells at D0 and D7 following prolonged culture, probed with indicated antibodies. **f** IB probed with the indicated antibody using lysate from MEFs 24 h following treatment with recombinant CYPA (10 ng/mL) and/or S100A4 (50 ng/mL). **g** IB probed with indicated antibodies using the 10–30 kDa fraction of CM following immunodepletion (ID) with anti-S100A4 (upper) or anti-CYPA (lower); ID with anti-IgG used as control. **h** IB using lysate from WI-38 cells incubated for 24 h with RM or CM that has undergone ID with the indicated antibody. IB probed with indicated antibodies. **i** Quantification of β-gal positive WI-38 cells 3 days following treatment with CYPA (10 ng/mL) and S100A4 (50 ng/mL). Data represent mean value of three biological samples ± SEM. *P < 0.05 (two-tailed *t* test). Blots are representative of at least two biologically independent experiments. Analysis of fold-change normalized to control lane shown below IB where indicated

We next wanted to identify specific factors in the CM that promote the increase in p52. Exposure of CM to proteinase K completely blocked the ability of CM to induce p52 suggesting that one or several proteins mediate the effect (Fig. 4a). To identify the potential protein(s) involved in an un-biased manner, we used a size-exclusion approach. CM was concentrated and then passed through progressively finer filters to obtain samples of different molecular weights which were subsequently applied to MEFs (Fig. 4b). Only the 10–30 kDa fraction was able to increase p52 abundance to a level approaching that seen with the whole CM (Fig. 4c). MEFs treated with fractions containing proteins greater than 30 kDa in size failed to affect p52 a finding also seen with proteins < 10 kDa. The 10–30 kDa fraction was run on polyacrylamide gel matrix and the single visualized band cut out and analyzed by liquid chromatography/mass spectrometry (LC-MS/MS). 11 candidate proteins were identified (Additional file 2: Table S1). Among these, two stood out because they are between 10 and 30 kDa, are known to be secreted factors and have previously been associated with aging: peptidylprolyl cis-trans isomerase A (Ppia, cyclophilin A, CypA) and S100 calcium-binding protein a4 (S100a4, mts1). To further validate the presence of these proteins in the media, we examined CM and found that in the 10–30 kDa fraction there is substantially higher amounts of both proteins than in RM (Fig. 4d). In addition, in human WI-38 cells, long-term culture leads to a substantial increase in the abundance of both S100A4 and CYPA (Fig. 4e).

To determine whether these proteins can induce p52, we first estimated the amount of each protein in the 10–30 kDa fraction by titrating exogenous purified protein to that in the CM (Additional file 1: Fig. S2a). Next, we incubated MEFs with active recombinant CYPA and S100A4, each at the concentration estimated to be present in CM, and p52 abundance examined 24 h later. CYPA and S100A4, either alone or together, induce an increase in p52 without altering the level of p50 (Fig. 4f). These data suggest that S100A4 and CYPA are both sufficient to induce p52. Next, to examine the requirement of these proteins for CM to induce p52, we used immunodepletion (ID) to remove each protein from the media. Pre-incubation of CM with the indicated antibody substantially reduced the amount of each protein in the CM without affecting the abundance of the other protein (Fig. 4 g). Moreover, ID of either protein substantially blocked the ability of CM to induce p52 in WI-38 cells (Fig. 4 h). To study whether these proteins promote cellular senescence, we examined β-gal staining in the presence of recombinant S100A4 and CYPA. Notably, when these proteins were added, there was a significant increase in the percent of β-gal positive cells (Fig. 4i). Together, these findings indicate that CYPA and S100A4 are sufficient to induce both increased p52 and cellular senescence and moreover, that their presence is required for CM to efficiently increase p52.

### CYPA and S100A4 induce p52 via STAT3 signaling

To study the mechanism linking CM to the induction of p52, we used a series of inhibitors that target pathways known to mediate p52 activation. A substantial decrease in p52 activation was seen in the presence of the STAT3 inhibitor WP1066 [25] (Additional file 1: Fig. S2b), an observation supported by the finding that G06976, a PKC inhibitor that has significant effects against JAK2/STAT3 [26, 27] also blocked up-regulation of p52 whereas the PKC inhibitor (staurosporin) did not. These results provided preliminary data that STAT3 might be involved in upregulation of p52 by CM.

**Fig. 5:**
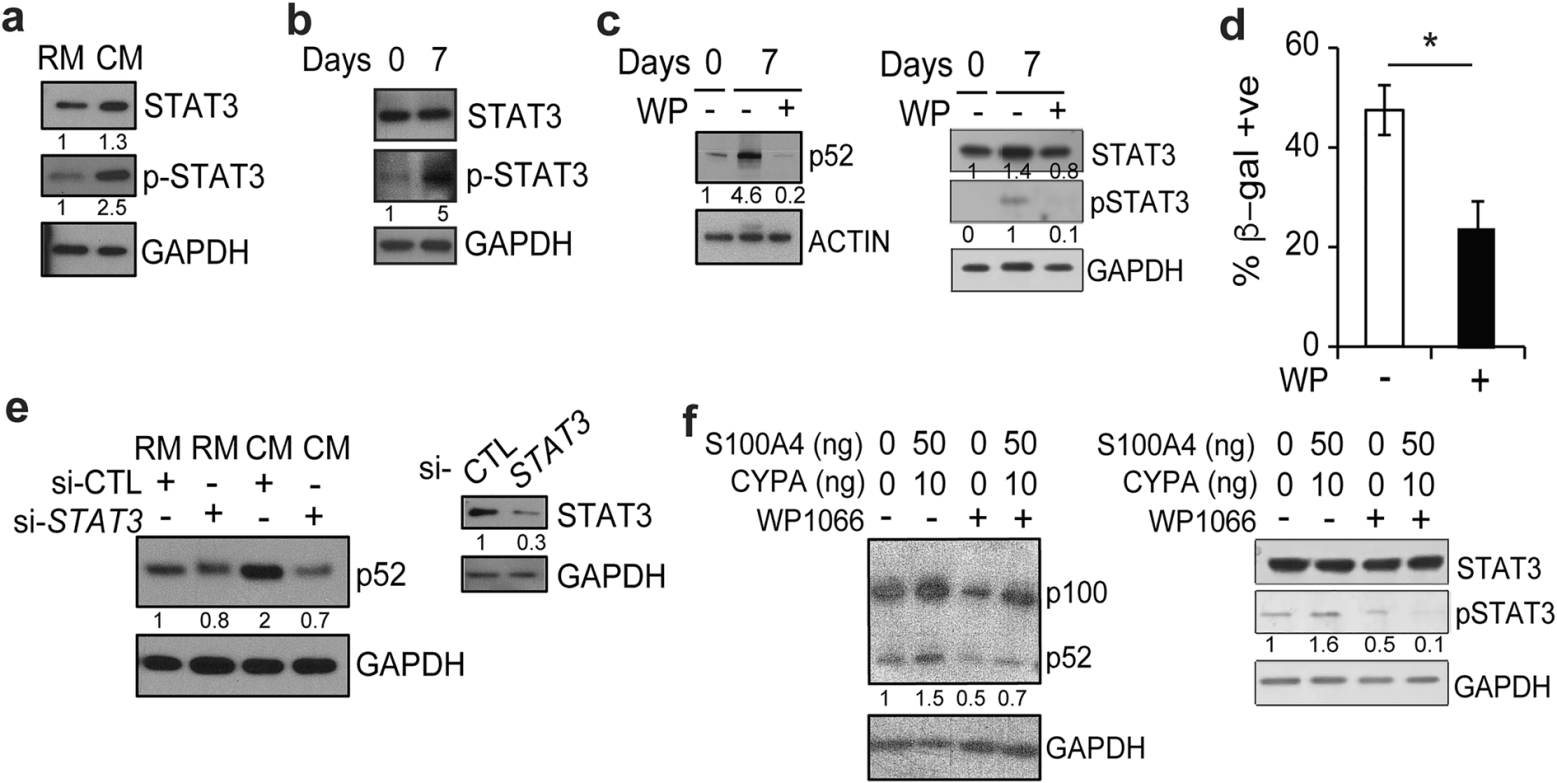
CYPA and S100A4 induce p52 via STAT3 signaling. **a** Immunoblot (IB) using WI-38 cells incubated in regular media (RM) or conditioned media (CM) for 24 h, probed with the indicated antibodies. P-STAT3 (phospho-STAT3). **b** IB using lysate from WI-38 cells harvested on Day 0 and 7 following prolonged culture, probed with indicated antibodies. **c** IB using lysate from WI-38 cells at D0 and D7 treated with or without WP1066 (WP, 5 µM). **d** Quantification of β-gal-positive WI-38 cells on Day 7 following prolonged culture ± WP1066 (5 µM). Data represent mean value of three biological samples ± SEM. *P < 0.05 (two-tailed *t* test). **e** IB using lysate from WI-38 cells transfected with si-*STAT3* or si-control (CTL) and incubated with either RM or CM for 24 h. Inset: IB of same cells with indicated antibody following transfection with siRNA. **f** IB using lysate from WI-38 cells incubated in recombinant CYPA or S100A4 for 24 h with or without WP1066 (5 µM). Blots are representative of at least two biologically independent experiments. Analysis of fold-change normalized to control lane shown below IB where indicated

To study whether STAT3 is involved in the up-regulation of p52, we incubated WI-38 cells with CM and noted an increase in phosphorylated STAT3 in the presence of CM (Fig. 5a). Phospho-STAT3 is also increased in cells maintained in culture for 7 consecutive days (Fig. 5b). To examine whether STAT3 mediates the increase in p52, we treated cells with the STAT3 inhibitor, WP1066, and found that it completely blocked the increase in p52 induced by long-term culture (Fig. 5c). In addition, WP1066 also significantly decreased the percentage of β-gal positive cells at Day 7 (Fig. 5d), highlighting the senescence-inducing role of STAT3 [28]. To further examine the role of STAT3 in this pathway, we used siRNA to specifically reduce *STAT3* expression and found that knock-down of *STAT3* substantially blocked the increase in p52 and reduced the increase in β-gal positive cells induced by CM (Fig. 5e and Additional file 1: Fig. S2c). Finally, to examine whether STAT3 is involved in promoting the p52 response to CYPA and S100A4, we incubated WI-38 cells with recombinant CYPA and S100A4 in the presence of WP1066. Addition of WP1066 completely blocked the increase in p52 induced by the recombinant proteins (Fig. 5f). These data suggest that CYPA and S100A4 in CM induce p52 via a pathway involving activation of STAT3.

### Senescent cells have upregulated p52 chromatin enrichment

**Fig. 6:**
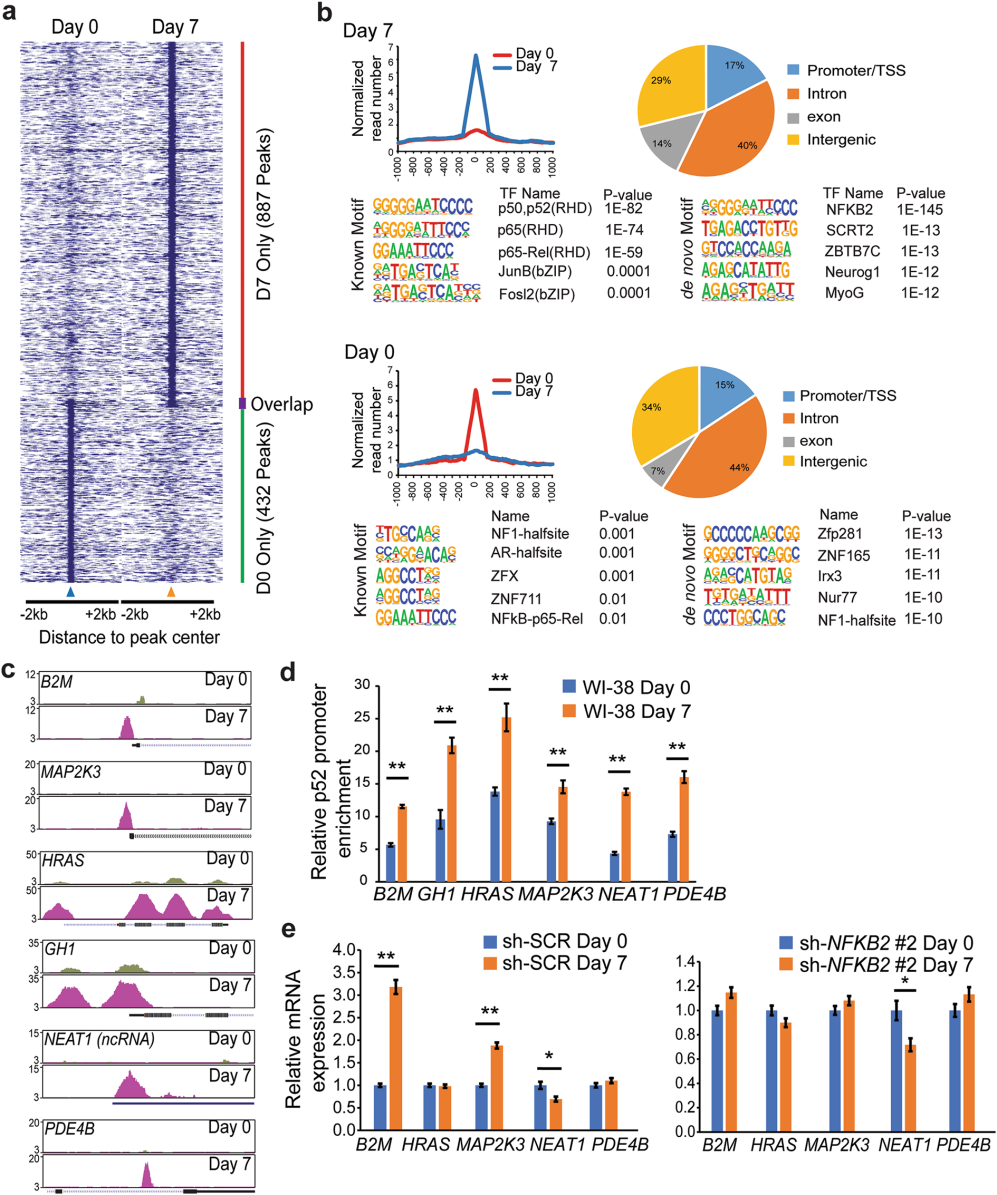
Senescent cells upregulate p52 Chromatin enrichment. **a-c** ChIP-seq analysis of WI-38 cells at D0 and D7 following prolonged culture. **a** Heatmap of differential binding of endogenous p52 to gene promoters. **b** Histograms demonstrating average peak intensities of ChIP-Seq data from WI-38 cells at Day 0 (lower) and Day 7 (upper). Pie charts demonstrate the distribution of p52 Chip-Seq peaks annotated to specific genomics features on indicated day. Primary binding motifs also shown. **c** UCSC genome browser view of p52 binding peaks at candidate gene regulatory elements on the indicated day. **d** ChIP-qPCR analysis of p52 chromatin enrichment at indicated gene on day 0 or 7 following culture of WI-38 cells. Data show mean enrichment at specific gene relative to input and IgG control, ± SEM of two independent experiments. **e** qPCR analysis of indicated genes in WI-38 cells expressing sh-RNA targeting *NFKB2* (right) or a scrambled sequence (SCR, left). Data show mean expression relative to *GAPDH* normalized to day 0, ± SEM of three independent experiments. *P < 0.05. **P < 0.01 (two-tailed *t* test)

p52 mediates its effects by interacting with chromatin and modulating gene expression. Given the increase in nuclear p52 with prolonged culture, we examined the genome-wide changes in p52 chromatin enrichment following extended culture. WI-38 cells were cultured for 7 days and enrichment of endogenous p52 was assessed by ChIP-sequencing. On day seven, p52 was bound to twice as many chromatin sites as at baseline (Fig. 6a and GSE182248). Analysis of binding sites demonstrate that the largest group of sites are intronic and that the majority of binding sites correlate with p52 and NF-κB consensus motifs (Fig. 6b). Several genes whose regulatory elements were enriched with p52 on day 7 have been closely linked to aging and senescence including *B2M*, *GH1* and *MAP2K3* (Fig. 6c and Additional file 1: Fig. S2d). To validate the change in p52 chromatin enrichment at these genes, we used ChIP-qPCR in WI-38 cells harvested on day 0 and 7. Using primers that span a region within the p52 binding peaks, we find that p52 is significantly enriched at the regulatory regions of these genes on day 7 compared to day 0 (Fig. 6d). Finally, to study the expression of these potential p52-targets, we isolated total mRNA from cells on day 0 and day 7 and examined the expression of specific genes by qPCR. Only the expression of *B2M* and *MAP2K3* was significantly elevated on day 7 compared to day 0, while *NEAT1* was decreased on day 7 (Fig. 6e). Notably, although p52 was significantly enriched to chromatin surrounding *HRAS* and *PDE4B*, their expression was not altered on day 7. Knockdown of p52 using two independent sh-RNAs, reversed the changes in *B2M* and *MAP2K3* expression on day 7 compared to day 0 (Fig. 6e and Additional file 1: Fig. S2e) highlighting the requirement of p52/NFKB2 for their expression change. These results demonstrate that the senescence-associated increase in nuclear p52 is reflected by increased chromatin recruitment of p52 that results in changes in expression of specific genes.

## Discussion

p52, the mature protein product of *NFKB2*, is formed under tight physiological control. In the current work, we observe that senescent cells have substantially elevated nuclear p52 compared to young/pre-senescent cells. Using a combination of over-expression and inhibition, we demonstrate that p52 is necessary for efficient induction of senescence. Specifically, we find that expression of mature p52 results in increased cellular senescence, a finding not seen with expression of either p100 or p50. The increase in senescence is only evident with expression of wildtype p52 but not mutant p52 that is unable to bind DNA. To further examine this finding, we incorporated loss of function studies. While genetic depletion of p52 is hampered by the concomitant loss of p100, by incorporating the peptide inhibitor, SN52, that blocks p52 nuclear translocation without altering p100, we were able to study p52 inhibition independent of p100. Notably, SN52 has its own peptide control and use of this peptide mitigates the pleiotropic effects seen with chemical NIK and IKK inhibitors. SN52 both decreased nuclear accumulation of p52 and reduced cellular senescence. These findings strongly support the hypothesis that elevated nuclear p52 actually promotes senescence and is not just an epi-phenomenon of senescence.

While NF-κB signaling has been extensively linked to senescence and formation of the SASP [5, 29], an association between senescence and p52 has been rarely examined. Studies that have looked at p52 and senescence have only used loss of function experiments targeting *NFKB2*. While one study reported that *NFKB2* blocked stress- and oncogene-induced senescence [30], in a separate report, knockdown of *NFKB2* was noted to increase senescence [21]. In addition, an independent investigation found that loss of *NFKB2* was indirectly associated with senescence [31]. Although these reports all suggest that NF-κB2 reduces senescence, the opposite of our finding, none actually examined mature p52 itself. Importantly, it is well recognized that p52 has distinct actions to its parental protein, p100. While p52 generally blocks cell death and promotes malignancy, p100 acts as an apoptotic factor and has tumor suppressive properties [15, 17, 18]. Notably, the presence of ankyrin repeats in p100 enable it to behave as an inhibitor-κB (IκB) protein that sequesters NF-κB dimers [32]. In the current study, we targeted p52 independent of p100 and found that unlike the results with *NFKB2* manipulation, mature p52 induces and is required for efficient cellular senescence.

In examining the mechanism by which p52 is activated in senescing cells, we observed that proteins present in conditioned media promote the increase in p52. Size exclusion filtration identified factors between 10 and 30 kDa in size as being required for the effect on p52 and MS/MS analysis identified S100A4 and CYPA as two proteins released by senescing cells that promote the increase in p52. While each protein was able to induce p52 on its own, the effect of the two proteins together was less than additive. It is suspected that this finding was due to both factors activating p52 via similar downstream signaling responses involving STAT3. Notably, both CYPA and S100A4 are closely linked to organismal aging [33–35]. Previous work has also shown that S100A4 induces nuclear translocation of p52, a finding blocked by the extracellular domain of the receptor for advanced-glycation-end-products (ex-RAGE) [36]. Mechanistic studies revealed that conditioned media induced p52 via a pathway involving phosphorylation of STAT3, a response that was blocked by the STAT3 inhibitor WP1066 and by knockdown of *STAT3*. Together these findings identify a pathway by which S100A4 and CYPA, released by cells in culture, induce p52 activation via STAT3 that further promotes senescence. Notably, this pathway is consistent with a prior report demonstrating a role for STAT3 in activation of p52 [15].

Given that p52 modulates its effects via interaction with chromatin and DNA binding, we examined genome-wide changes in p52 chromatin enrichment. A substantial increase in the DNA binding of p52 was seen in senescent cells compared to baseline. This increase is consistent with the increase in nuclear p52 protein seen in aged tissue. Subsequent examination of the expression level of the genes bound by p52 demonstrated that while increased p52 binding was associated with increased expression of some factors (e.g. *B2M* and *MAP2K3*), the increased binding to *NEAT1* was associated with decreased gene expression. From a mechanistic perspective, although changes in expression may be due to factors unrelated to p52, the inhibitory effect of p52 can be explained by the fact that p52 lacks a transactivation domain (TAD) and its homodimers can be inhibitory, whereas when p52 dimerizes with RELB and p65 or when associated with co-regulators like BCL-3, it can activate transcription [37, 38]. Among the p52 targets that we identified, both B2M (β2-microglobulin) and MAP2K3 (p38 kinase) are closely linked to cellular senescence and aging. β2-microglobulin has been shown to decrease cognitive function and promote hippocampal dysfunction [39], while MAP kinases, including the target of MAP2K3, p38, is a central factor in the SASP [40, 41].

A central observation from the current work and our prior report [20], is that nuclear p52 is elevated in senescent/aged mammalian tissue. When taken with the p52 manipulation studies, these findings support the hypothesis that elevated p52 contributes to cellular senescence and mammalian aging. Notably, by examining cells at different time points and passages, we find that there is a gradual increase in p52 abundance over time and that even exposure to CM for 24 h is sufficient to increase p52. Whether there is a specific level of p52 that is necessary to induce senescence is unclear. However, given that nuclear p52 abundance is tightly-regulated and maintained at a low level, it is likely that sustained elevation of nuclear p52, seen with prolonged culture or in aged cells, is necessary for inducing, or enabling, the senescence response. Whether there is a specific duration of elevated p52 required to promote irreversible changes that result in senescence remains unclear. Finally, although we find that elevated p52 is sufficient to induce senescence, physiologically the increase in p52 seen in cultured cells is induced by factors secreted by these same cells. Together, these observations suggest that the increase in p52 and the senescence response are cyclically linked such that factors released into the extracellular space induce the increase in p52 that then promotes further alterations that underlie the senescent phenotype.

In general, the data are consistent with the overall propensity of p52 to promote inflammation and malignancy [42], central features of the aging phenotype. Interestingly, further support for such a role of p52 is seen in the observation that when mature p52 was constitutively expressed in mice, there was an increase in inflammation and overall animal mortality [43].

## Conclusions

It is recognized that the NF-κB pathway plays a central role in senescence and aging. One of the most striking observations of aged tissue with respect to NF-κB is the substantial increase in mature p52 protein. While it may be argued that this increase is a result of senescence, the data from the current report demonstrate that p52 induces senescence and, given the importance of cellular senescence to aging, our results suggest that increased p52 promotes organismal aging. In addition, the finding that p52 is induced by factors in conditioned media supports the known paracrine components of the senescence program in that factors released by aging cells act to further induce senescence. These data would suggest that targeting p52, but not p100, may be a potential senolytic focus that can be developed to modulate mammalian aging and chronic disease.

## Methods

### Cell lines and reagents

WI38 cells were purchased from American Type Culture Collection. Cells were cultured in DMEM supplemented with 10% FBS (R&D Systems) and 1% Penicillin-Streptomycin (ThermoFisher Scientific). These cells were screened for mycoplasma every four months using the ATCC Universal Mycoplasma Detection Kit (Catalogue # 30–121 1012 K). Early passage primary MEFs were harvested and cultured from C57BL/6 mice as previously described [20, 44, 45]. β-gal staining was achieved using the Cellular Senescence Kit (Millipore). Recombinant proteins included Cyclophilin A (R&D) and S100A4 (Abcam). SN52 peptide (AAVALLPAVLLALLAPVQRKRRKALP) and SN52-mut peptide (AAVALLPAVLLALLAPVQRNGRKALP) were acquired form GenScript. Inhibitors used were: WP1066, BMS345541, G06976, Staurosporine, and KU60019 (all from Selleck), and Roscovitine (Sigma). The following siRNAs were used: siGENOME non-targeting siRNA#3 (Dharmacon) and si-*STAT3* (sc-29,493, Santa Cruz). siRNA transfection was performed with Oligofectamine (Invitrogen).

### Population doubling analysis

Population doublings (PD) were analyzed beginning with Passage 2 (population estimated to already have undergone 3 PDs). Cells were plated onto 6 well plates at 25% confluency. Once cells reach a confluency of ~ 75% (equal to 2 PDs), they were once again passaged and plated onto 6 well plates at 25% confluency. This was repeated until cells slowed down in PDs and looked unhealthy.

### β-Galactosidase quantification

Primary MEFs from the indicated passage were plated onto 6-well plates at about 60% confluence and fixed with formaldehyde for 15 min at room temperature and then kept in PBS until further use. Streptadivdin (SA) β-galactosidase staining was done per kit instructions (Millipore). Briefly, cells were incubated with freshly made SA β-gal Staining Solution containing X-gal overnight at 37 °C, without CO2 and protected from light. Plates were then rinsed in PBS and allowed to air dry. To determine senescence, the percentage of cells positive for β-gal was calculated from a total of at least 200 cells imaged per plate. β-gal quantification was performed using three biological repeat samples. Representative images are displayed.

### Immunoblotting and antibodies

Immunoblots were performed as previously described [46] with the following primary antibodies: anti-GAPDH (sc-32,233, Santa Cruz), anti-p100/p52 (4882 or 37,359 S, Cell Signaling), anti-p105/p50 (D4P4D, Cell Signaling), anti-lamin (4777 S, Cell Signaling) anti-p65 (D14E12, Cell Signaling), anti-RelB (10,544 S, Cell Signaling), anti-IKKα (2682 S, Cell Signaling), anti-NIK (4994 S, Cell Signaling), anti-HA (sc-7392, Santa Cruz), anti-Actin (sc-8432, Santa Cruz), anti-CYPA (sc-134,310, Santa Cruz), anti-S100A4 (13,018 S, Cell Signaling), anti-Histone H3 (sc-517,576, Santa Cruz), anti-STAT3 (sc-482 X, Santa Cruz), and anti-phospho-STAT3 (sc-8059, Santa Cruz). All immunoblots are representative of at least two separate experiments and representative images displayed.

### Quantification of blots

Semi-quantitative analysis of IBs was performed using ImageJ (v1.52a) gel analysis tool set, normalized to loading. All quantification data are representative of at least two independent biological experiments.

### Cell fractionation

Cell fractionation was performed as described before [46]. Briefly, cells were harvested and resuspended in lysis buffer (20 mM Tris-HCl, pH 7.5, 10 mM KCl, 1.5 mM MgCl2, 1% Triton X-100 and protease inhibitor cocktail) on ice for 10 min. Triton-X was added to the mixture for a final concentration of 0.5% and vortexed for 15 s. Cell lysate was then centrifuged at 1000 g for 3 min, and cleared by centrifugation at 15,000 rpm for 10 min. The cytoplasmic fraction was collected, while the nuclear pellet was lysed in nuclear lysis buffer conditioned with protease inhibitors. Experiments were performed at least twice.

### RNA isolation and real time qPCR

Total mRNA was isolated using Trizol reagent (Ambion) or with RNA mini-prep kit (Zymo). Quantitative real-time PCR (qPCR) was performed using EvaGreen SYBR Green (BullsEye) as described previously [47]. qPCR primers can be found in Additional file 2: Table S1. Whole cell mRNA analysis was performed by first normalizing raw Ct values to *GAPDH* expression, and then to control samples. Experiments were performed using biological triplicate samples.

### Immunofluorescence (IF) staining

Cells were plated on chamber slides and fixed with paraformaldehyde for 10 min. After blocking with 5% BSA in 0.1% Triton, slides were incubated overnight at 4 °C with the following primary antibodies: anti-p100/p52 (4882 S, Cell Signaling) and anti-γ-H2AX (9718, Cell Signaling). Slides were rinsed with TBS, incubated with Alexa Fluor 488 secondary antibody (Life Technologies) for 2 h at room temperature, and then rinsed and cover-slipped with mounting medium containing DAPI (Fisher Scientific). For negative controls, primary antibody was replaced with goat serum.

Fluorescence images were captured on a Zeiss Axiovert 200 M microscope. DAPI and AlexaFluor 488 images were captured using sequential acquisition to give separate image files for each. At least three high power fields (20x-40x) were selected by viewing DAPI staining. This approach provided data on at least 200 cells. Quantification was calculated from samples plated in triplicate by counting all positive cells in each field per run and averaging their total number. The percentage of these among the total cells was then reported. Each experiment was repeated three times.

### Conditioned media harvest, size filtration and MS/MS

MEFs and WI-38 cells were cultured in a 150-mm plate and maintained in DMEM supplemented with 10% FBS (R&D Systems) until confluent. Cells were rinsed with PBS and incubated in DMEM without FBS for 24 h and conditioned media collected. The media was then filtered to remove debris and detached cells and concentrated based on protein size using Amicon Ultra centrifugal units (Millipore) with filters of varying sizes (10, 30, and 50 kDa) per manufacturer’s instructions. Where indicated, concentrated fractions were run on SDS-PAGE and analyzed by silver staining using the Silver Stain Kit (Biorad). Single visualized bands were cut out and sent out for mass spectrometry analysis by LC-MS/MS.

### Lentiviral production and infection

Recombinant lentiviral particles were produced with the lentiviral expression plasmid pLKO0.1 using a scrambled shRNA sequence or two independent sequences targeting *NFKB2* (Additional file 2: Table S1). shRNA constructs were mixed with packaging plasmids, psPAX2 and pMD2.G, and transfection were performed using TransIT-LT1 Transfection Reagent (Mirus Bio). Briefly, 8 × 10^5^ HEK293T cells were cultured in 10-cm plates with Opti-MEM (ThermoFisher Scientific) and transfection performed when the cell density reached 50–60% confluency. Six hours post transfection, the culture medium was replaced with fresh DMEM/10% FBS (Atlanta Biologicals) and 48 h later the medium containing lentivirus was collected and centrifuged at 4000 × g at 4 °C for 10 min to remove cellular debris. The supernatant was filtered through a 0.45 μm filter and then concentrated with Lenti-X concentrator (Clontech). For lentivirus infection, target cells were seeded at 50% confluency and infected with lentiviral particles at multiplicity of infection (M.O.I.) of 5, in the presence of 8 µg/ml polybrene (Sigma-Aldrich). Primary cells were subsequently used for downstream experiments.

### Chromatin immunoprecipitation assay

ChIP followed by quantitative real-time PCR (qPCR) was performed using WI-38 cells. On the indicated day, cells were fixed with 1% formaldehyde for 10 min. Chromatin DNA was sonicated and clarified at 20,800 ×g at 4 °C. Supernatant from 2 × 10^6^ cells was used for each ChIP assay using Dynabeads Protein G (ThermoFisher Scientific). Ten microliter of ChIP-grade anti-p52 antibody (#37,359, Cell signaling) was used per ChIP. The immunoprecipitated DNA was amplified by real-time PCR. Primer sequences and amplicon positions are shown in Additional file 2: Table S1. Relative enrichment of p52 at the promoter region of each candidate gene was expressed as the percentage of the corresponding input sample (ΔCT). Experiments were performed with two biological replicates.

### ChIP-Seq library construction and data analysis pipeline

Chromatin DNA immunoprecipitation was performed as described above using the indicated antibody. ChIP-Seq libraries were constructed with NEBNext Ultra II DNA Library Prep Kit for Illumina (NEB). Libraries were then sent to Genomics Core Facility at the University of Chicago for quality control fragment analysis and sequencing with HiSeq4000 platform. Single-end 50 bp short reads were retrieved from the Genomics Core Facility. Samples were first trimmed with Trimmomatic (v0.39) to filter low-quality reads. Duplicated reads generated from PCR amplification were discarded with Broad Institute Picard Tools (v 2.25.7). The short reads were mapped to human genome (hg38) with Bowtie2 (v2.4.4), and sorted with Samtools (v1.13). Homer pipeline (v4.11) was used for peak calling (findPeaks) and annotation (annotatePeaks.pl). the false discovery rate (FDR) was set as default (0.001). The bedGraph file used for displaying peaks within UCSC Genome Browser was generated with the makeUCSCfile package from Homer. Heatmaps for binding around peak centers and histograms for average peak intensity were plotted with the annotatePeaks module in Homer suite. The libraries from input DNA were used as controls to enable removal of background random clusters of reads. Robust peaks with peak score ≥ 10 were used to consolidate peaks to the nearest genes.

### Statistical analysis

In vitro and other studies where indicated were analyzed by two-tailed Student’s *t* test with significance taken as *P* < 0.05.

## Supplementary Information

**Additional file 1.** p52 Aging RESUB.

**Additional file 2.** Table S1.

## Data Availability

Data generated in this study have been submitted to the NCBI Gene Expression Omnibus (GEO; http://www.ncbi.nlm.nih.gov/geo/) under accession number GSE182248.
