# Supplementary material for: p52 signaling promotes cellular senescence
Source: Cell Biosci. 2022 Apr 4;12:43. doi: 10.1186/s13578-022-00779-6 (PMC8981737; doi:10.1186/s13578-022-00779-6)
Supplement: Supplementary file 1 — Additional file 1. p52 Aging RESUB. [file 13578_2022_779_MOESM1_ESM.docx]

**xAdditional File 1: Figures S1 and S2**

**p52 signaling promotes cellular senescence**

Giovanna M Bernal, Longtao Wu, Ralph R Weichselbaum and Bakhtiar Yamini.


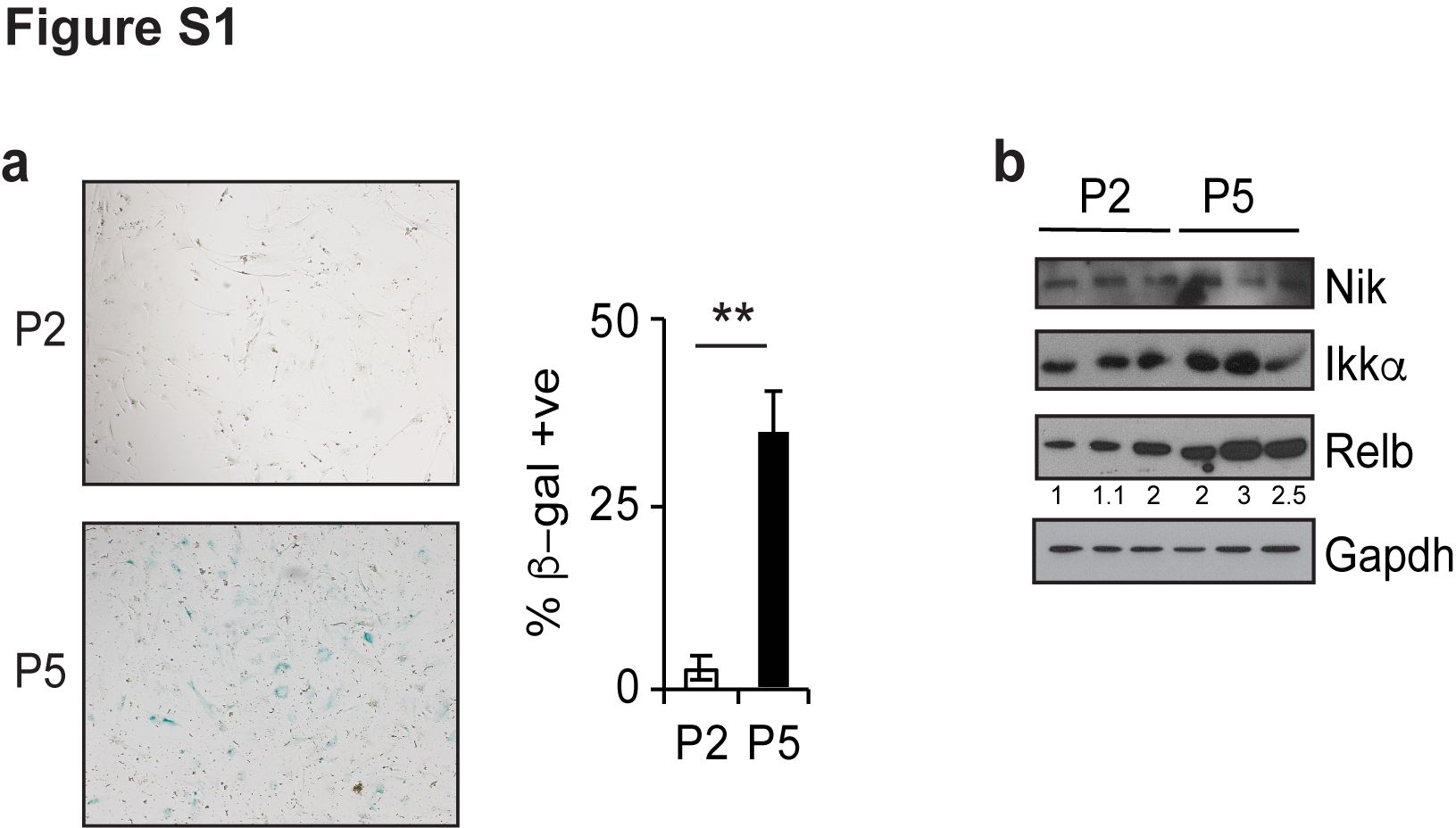


**Figure S1.** **a** Quantification of β-gal staining of primary MEFs at indicated passage. Representative images (left), quantification (right). Data represent mean value of triplicate biological samples, ± SEM. ***P* < 0.01 (two-tailed *t* test). **b** IB using whole cell lysate from MEFs at indicated passage probed with anti-NIK, anti-IKKα, anti-Relb and anti-Gapdh. Blots are representative of at least two biologically independent experiments. Analysis of fold-change normalized to control lane shown below IB where indicated.

**
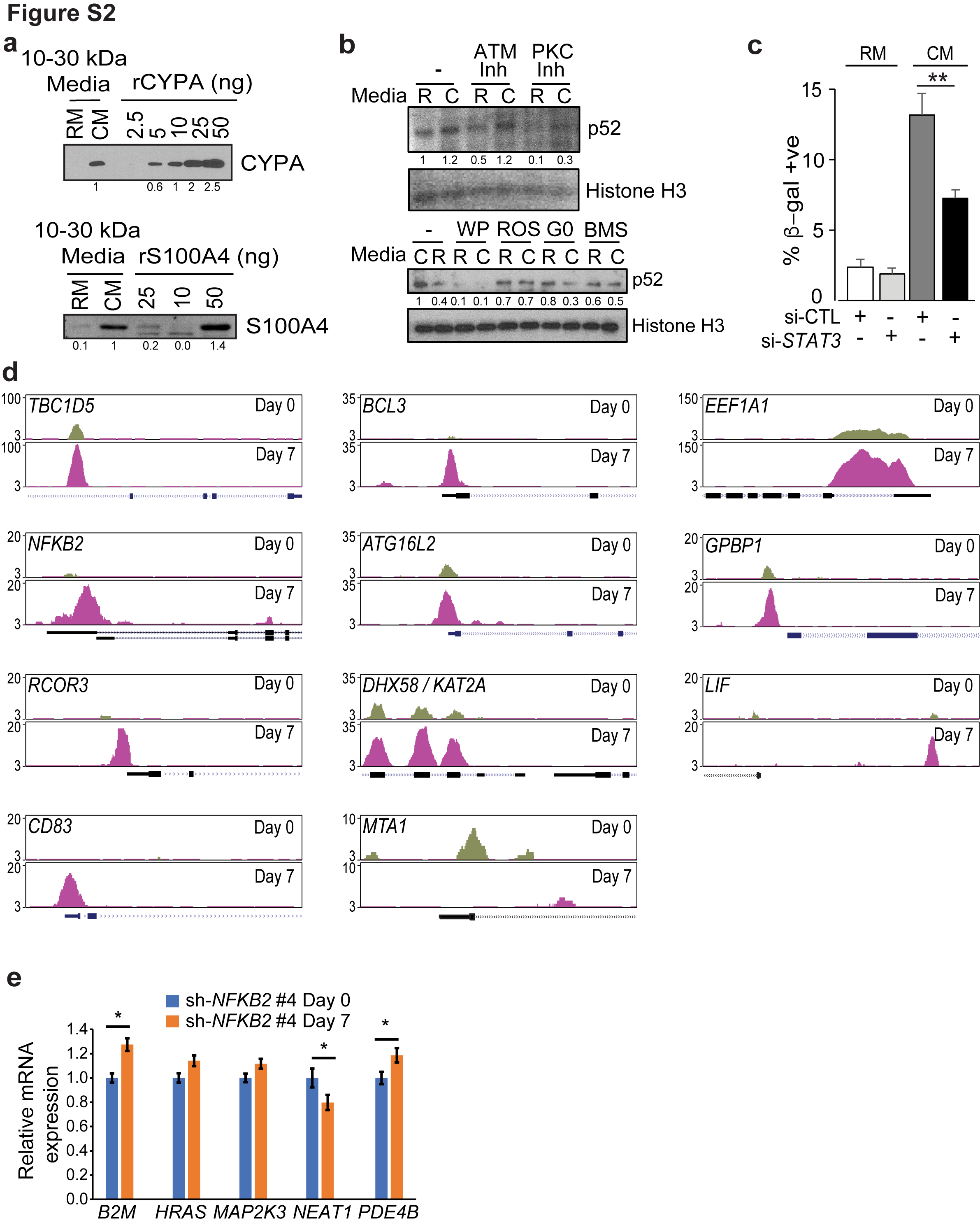
**

**Figure S2.** **a** IB using the 10-30 kDa fraction from regular media (RM) and conditioned media (CM) and indicated amount of recombinant CYPA or S100A4 protein. IB performed with anti-CYPA and anti-S100A4 as shown. **b** IB using nuclear extract from WI-38 cells following incubation with regular media (R) or conditioned media (C) and treatment with one the following inhibitors: WP1066 (WP), roscovitin (ROS), G06976 (G0), BMS345541 (BMS, IKK inhibitor), staurosporin (PKC Inh), and KU60019 (ATM Inh). IB was performed with anti-p52 and anti-Histone H3. **c** Quantification of β-gal-positive WI-38 cells transfected with si-*STAT3* or si-control (CTL) and incubated with either RM or CM for 5 days. Data represent mean value of three biological samples ± SEM. ***P* < 0.01 (two-tailed *t* test). **d** UCSC genome browser view of p52 binding peaks at candidate gene regulatory elements. **e** qPCR analysis of indicated genes in WI-38 cells expressing an sh-RNA targeting *NFKB2*. Data show mean expression relative to *GAPDH* normalized to day 0, ± SEM of three independent experiments. **P* <  0.05 (two-tailed *t* test). Blots are representative of at least two biologically independent experiments. Analysis of fold-change normalized to control lane shown below IB.
